# Characterization of integrated prophages within diverse species of clinical nontuberculous mycobacteria

**DOI:** 10.1186/s12985-020-01394-y

**Published:** 2020-08-17

**Authors:** Cody Glickman, Sara M. Kammlade, Nabeeh A. Hasan, L. Elaine Epperson, Rebecca M. Davidson, Michael Strong

**Affiliations:** 1 Center for Genes, Environment, and Health, National Jewish Health, Denver, CO USA; 2 Computational Bioscience Program, University of Colorado, Anschutz Medical Campus, Aurora, CO USA

**Keywords:** Mycobacteriophage, Nontuberculous mycobacteria, Prophage, Virulence, Growth rate

## Abstract

**Background:**

Nontuberculous mycobacterial (NTM) infections are increasing in prevalence, with current estimates suggesting that over 100,000 people in the United States are affected each year. It is unclear how certain species of mycobacteria transition from environmental bacteria to clinical pathogens, or what genetic elements influence the differences in virulence among strains of the same species. A potential mechanism of genetic evolution and diversity within mycobacteria is the presence of integrated viruses called prophages in the host genome. Prophages may act as carriers of bacterial genes, with the potential of altering bacterial fitness through horizontal gene transfer. In this study, we quantify the frequency and composition of prophages within mycobacteria isolated from clinical samples and compare them against the composition of PhagesDB, an environmental mycobacteriophage database.

**Methods:**

Prophages were predicted by agreement between two discovery tools, VirSorter and Phaster, and the frequencies of integrated prophages were compared by growth rate. Prophages were assigned to PhagesDB lettered clusters. Bacterial virulence gene frequency was calculated using a combination of the Virulence Factor Database (VFDB) and the Pathosystems Resource Integration Center virulence database (Patric-VF) within the gene annotation software Prokka. CRISPR elements were discovered using CRT. ARAGORN was used to quantify tRNAs.

**Results:**

Rapidly growing mycobacteria (RGM) were more likely to contain prophage than slowly growing mycobacteria (SGM). CRISPR elements were not associated with prophage abundance in mycobacteria. The abundance of tRNAs was enriched in SGM compared to RGM.

We compared the abundance of bacterial virulence genes within prophage genomes from clinical isolates to mycobacteriophages from PhagesDB. Our data suggests that prophages from clinical mycobacteria are enriched for bacterial virulence genes relative to environmental mycobacteriophage from PhagesDB.

**Conclusion:**

Prophages are present in clinical NTM isolates. Prophages are more likely to be present in RGM compared to SGM genomes. The mechanism and selective advantage of this enrichment by growth rate remain unclear. In addition, the frequency of bacterial virulence genes in prophages from clinical NTM is enriched relative to the PhagesDB environmental proxy. This suggests prophages may act as a reservoir of genetic elements bacteria could use to thrive within a clinical environment.

## Introduction

Nontuberculous mycobacterial (NTM) infections can cause serious pulmonary disease that may become chronic and affect quality of life even leading to death [1]. Many of the mycobacterial species that cause NTM infections are ubiquitous in the environment and are known to thrive in built environments, including premise plumbing [2–4]. The mechanism by which these organisms, which have long been recognized as environmental, become clinical pathogens is an active area of research with prior studies exploring host susceptibility [5], geographic factors [6], and changes in genetic composition [7]. Mycobacterial species are categorized into two broad groups based on differing growth rates in culture; rapidly growing (RGM) and slowly growing (SGM) [8]. Of the six species/subspecies examined in this study *M. avium, M. chimaera,* and *M. intracellulare* are described as having a slow growth rate. *M. abscessus subsp. massiliense, M. abscessus subsp. abscessus, and M. abscessus subsp. bolletii* have a rapid growth rate [9].

Bacteriophages are viruses that infect bacteria and some are capable of transferring genetic material between bacteria through a process called transduction [10]. Mycobacteriophages are bacteriophages that target mycobacteria. Environmental bacteria are subject to external pressures to adapt their genomes through horizontal gene transfer, including bacteriophage transduction [10]. Temperate bacteriophages are capable of exhibiting both the lysogenic and lytic life cycles. The lysogenic cycle commonly involves a bacteriophage integrating genetic material into a bacterial genome and replicating in tandem until the integrated bacteriophage, also known as a prophage, transitions into a lytic life cycle. In addition to chromosomal integration, prophages can also exist within a host bacteria extrachromosomally [11]. During the lytic phase, the bacteriophage utilizes the bacterial cellular machinery to create new phage particles that are then released during bacterial cell lysis. Some of the newly created temperate phage particles package bacterial genes at a low frequency, which are subsequently transduced during a new infection [12]. This form of transduction is called specialized transduction, which is defined by the restriction of transducible bacterial genes to those flanking the integration site of the prophage. Another form of transduction, termed generalized transduction, occurs during the lytic phase when bacteriophages engaging in the headful packaging process include random pieces of the host bacterial DNA [13]. Both forms of transduction are thought to be rare events, however, given the immense number of bacteriophage-bacterial interactions, transduction events are estimated to occur at scale in the environment [14].

Virulence is a general term that describes a pathogen’s invasive power, ability to overcome host defenses, and the replication efficiency of a pathogen within a host [15]. Prior studies have explored both the role of bacteriophages to increase virulence in bacteria and the effect of bacteriophage resistance on reduced virulence [16, 17]. There is a selective advantage for bacteria that contain prophages with genetic elements capable of increasing propagative success and providing super-infection immunity [18]. It is possible that genes carried by mycobacteriophages during selective transduction events could impact virulence as seen in other bacteria such as *Escherichia coli*, *Vibro cholerae, Corynebacterium diphtheriae, and Streptococcus pyogenes* [19]. Prophages in these bacterial pathogens contain elements that contribute to quorum sensing, enzymatic functions, and extracellular toxicity [19, 20].

Here, we explore the frequency of integrated prophages in NTM genomes and characterize the composition of bacterial genes within predicted prophage elements. Genetic elements including tRNAs act as a potential insertion site for mycobacteriophages using a tyrosine-integrase [21]. Our hypothesis is that increased tRNA abundance is associated with an increased abundance of integrated prophages in mycobacteria due to there being more targets for integration with tyrosine-integrases. An important note is that other integrases can insert mycobacteriophages into genetic elements other than tRNAs and extrachromosomal prophages do not integrate at all [22]. CRISPR elements provide a bacterial defense mechanism against viral challenge, and research in *Cronobacter sakazakii* suggests CRISPR elements with fewer spacers are more susceptible to prophage integration [23, 24].

To explore differences between clinical prophages and environmental mycobacteriophages, we utilized PhagesDB, which is a data repository mostly composed of mycobacteriophages isolated from the environment [25]. A majority of these mycobacteriophages were identified using a phage plaque screening assay to identify lytic and temperate mycobacteriophages capable of infecting the non-pathogenic species *M.* s*megmatis*. Mycobacteriophages from PhagesDB are organized into sequence clusters, indicated by letters (A-Z), based first on sequence similarity and then shared functional protein families [26]. Lettered clusters typically exhibit similar lifestyle and functional behavior. Prior research has suggested PhagesDB mycobacteriophages clusters K, G, and A are capable of infecting clinical NTM including RGM and SGM [27, 28]. Our hypothesis is that prophages from genomes of clinical isolates may be enriched for bacterial virulence genes compared to environmental mycobacteriophages from PhagesDB. Exploring this hypothesis helps us to investigate the ability of prophages to act as a genetic repository for bacterial virulence genes within clinical NTM genomes.

## Methods

### Bacterial genome assembly and isolate datasets

In this study, we utilized two publically available datasets. First, we downloaded the complete genomes from two NTM species in the NCBI assembly database (Supplementary File 1), *M. abscessus* and *M. avium* (*n* = 53 complete genomes)*.* These species were selected because they represent the two most common NTM species of clinical significance [29]. All complete genomes were isolated from clinical sources, with the exception of an *M. avium* isolate from a hospital water source [30]. Our decision to look at complete genomes was driven by the desire to examine bacteriophage trends between species regardless of assembly status (complete vs. draft genome).

The second dataset includes a collection of 318 NTM isolates from 168 individuals diagnosed with cystic fibrosis (CF) [31]. All samples were cultured on solid media, converted to glycerol stock aliquots, and the DNA was extracted for whole genome sequencing using an optimized mycobacterial DNA preparation protocol [31, 32]. Bacterial isolates were collected in a longitudinal manner, however, only one isolate genome per patient per species was retained for this prophage analysis. Fourteen patients had multiple NTM species, and in these cases, one isolate from each species was retained (*n* = 182 draft genomes). This dataset includes six different mycobacterial species and subspecies: *M. abscessus subsp. massiliense, M. abscessus subsp. abscessus, M. abscessus subsp. bolletii, M. avium, M. chimaera,* and *M. intracellulare* (Table 1).

**Table 1:** Summary statistics of prophages predicted in the NTM draft genomes assemblies. Median counts of tRNA, N50 Lengths, and counts of contigs in NTM draft genomes are shown with the ranges in parenthesis. The edge case average is the total number of edge cases divided by the draft genome count

Species	Genomes Count	Genomes with Phage	Predicted Prophages	Median tRNA Count	Median N50 Length	Median Contig Count	Edge Cases Average
*M. avium*	43	1	1	57 (54–108)	81,670 (56262–116,826)	206 (126–336)	1.07
*M. chimera*	11	1	1	55 (49–81)	86,644 (78123–101,816)	193 (116–251)	1.00
*M. intracellulare*	23	3	4	52 (50–54)	101,938 (82877–131,830)	104 (83–145)	0.13
*M. abscessus*	76	42	51	48 (45–116)	168,941 (87141–327,102)	67 (41–191)	2.60
*M. bolletti*	4	3	3	48.5 (48–49)	208,710 (140476–226,296)	45 (37–59)	2.50
*M. massiliense*	25	11	12	51 (46–84)	215,865 (97542–413,240)	53 (39–143)	2.84

Paired-end reads from draft genomes in the CF dataset were assembled using Unicycler into contiguous sequences (contigs), known as draft genomes, with a median N50 ranging from ~ 82 kilobases to ~ 216 kilobases (Table 1) [33]. Numbers of contigs in the draft genome assemblies ranged from 37 to 336. Analysis of assembly completeness based on N50 length and the number of contigs metrics revealed three outliers, which were removed from downstream analysis. To understand if assembly fragmentation affected prophage prediction in our draft genomes, we explored the edge cases, which are defined as predicted prophages within 100 bases of either end of a contig. Given the distinctions in assembly statistics by growth rate, linear mixed modeling was performed to calculate statistical significance of assembly features against prophage frequency.

Species identifications of draft genomes were determined using a method of average nucleotide identity (ANI) as described previously [31] and sequence reads mapped to active reference genomes to generate phylogenies [34, 35]. Reads were mapped using Bowtie2 software [36] and single nucleotide polymorphisms (SNPs) were determined using Samtools mpileup [37]. Mycobacterial genotypes were concatenated and used to make phylogenetic trees using maximum likelihood with 1000 bootstrap replicates in Randomized Axelerated Maximum Likelihood-Next Generation (RAxML) [38]. SNP distances between groups in the tree file were used to perform PERMANOVA analysis in R. Phylogenetic trees were annotated and visualized with ggtree [39]. Additional tree file manipulations and visualizations were performed with ETE3 [40].

ARAGORN v1.2.38 was used to quantify the tRNAs in the mycobacterium [41]. CRISPR identification was performed using CRT with default parameters (minimum 3 repeats, minimum length 19 base pairs, maximum length 38 base pairs) [42].

### Integrated prophage discovery

Prophage discovery was performed using the agreement of two prophage discovery tools, Phaster and VirSorter [43, 44]. Phaster and VirSorter use different sequence similarity methods against known viruses to find prophage elements within bacterial genomes. Phaster was utilized because of the ability to consider the completeness of a putative prophage region through identification of elements such as attachment sites. VirSorter differs from Phaster in that it does not find attachment sites, however VirSorter outperforms other tools on prophage identification in fragmented genomic datasets [44]. A custom application programming interface (API) script was used to identify prophages from the Phaster web server, whereas VirSorter prophage identification was performed locally. The output of Phaster contains three confidence levels for a prophage prediction with “intact” possessing the strongest support, “questionable” having some support, and “incomplete” being the least confident. VirSorter also ranks prophage predictions into three numeric levels (4 strongest, 5 middle, and 6 low support) in addition to predicting individual contigs from the draft genomes as stand-alone viruses (1 strongest, 2 middle, and 3 low support). Confidence levels of Phaster predictions were manually set to the scale of VirSorter prophages. Only Phaster predicted prophages with overlapping ranges between the two tools were retained. Predicted prophages were further filtered by requiring identified attachment sites from Phaster, at least 10 proteins from the predicted prophage match against the PhagesDB protein database, and the presence of an integrase gene [45]. Edge case identifications incorporate all predicted prophages, not only those selected by both prophage prediction tools.

Dereplication of predicted prophage elements was performed using VSEARCH [46] to identify genetically identical prophages between different genomes, which may be evidence of transmission or contamination.

### Prophage identification and genome annotation

PhagesDB uses MMSEQ2 to cluster mycobacteriophage gene products into gene “phamilies”, then uses Splitstree to create functional clusters based on presence or absence of gene “phamilies” [47]. The version of PhagesDB used was filtered to only contain mycobacteriophages.

Our approach to assign predicted prophages to clusters began by calculating average nucleotide identity (ANI) using the MUMmer toolset against known mycobacteriophages from PhagesDB [48]. Per prior work, an ANI greater than 60% and with a genomic coverage of at least 50% would cluster a phage [49]. Following ANI clustering, we selected BLASTp because the gene “phamilies” are not included in the data API, and the parameters of MMSEQ2 cluster are not clearly defined in prior works [26]. A match was based on DIAMOND Blastp homology with default parameters (e-value <1e-12, query coverage > = 70, identity cut-off > = 70) against proteins from PhagesDB downloaded using the PhagesDB API [50]. A minimum of 5 protein matches from the predicted prophage to a cluster are required for cluster assignment, otherwise all unidentified prophages are assigned to no cluster. Gene products of predicted prophages were identified using Prodigal [51]. The aggregation of cluster identifications from gene hits was used to determine the projected cluster of the predicted prophage. The clustering of predicted prophages was visualized using RAWGraphs.io [52].

Bacterial genes within predicted prophages were annotated using Prokka [53] with an additional virulence gene and phage protein database combining VFDB, Patric-VF, and PhagesDB proteins added as a parameter [25, 53–55]. Proteins were predicted using Prodigal and subsequently annotated with a combination of DIAMOND BLASTp and HMMscan using default parameters [50, 51, 56]. Prodigal has a default minimum peptide length of 90 [51]. Pairwise significance testing, comparing the abundance of virulence genes in the PhagesDB and the predicted prophages, was performed using a z-score test for two population proportions with binary success defined as having any bacterial virulence gene in the genome.

Prophage counts by mycobacterial species were visualized using pandas (version 0.20.3) and matplotlib (version 2.1.0) [57, 58]. Pairwise significance testing comparing the abundance of prophages between species growth rate was performed using a z-score test for two population proportions with binary success defined as having a prophage with more than 10 gene matches against PhagesDB in the genome.

Pangenome analyses were performed using Roary [59] to identify core genes (present in > 95% of prophages) and shell genes (present in between 15 and 95% of prophages) amongst all ORFs in the predicted prophages (*n* = 96). In addition, assigned PhagesDB clusters of predicted prophages with more than five prophages per cluster were subjected to another pangenome analysis (*n* = 81).

## Results

### Abundance of prophages in NTM species

**Fig. 1:**
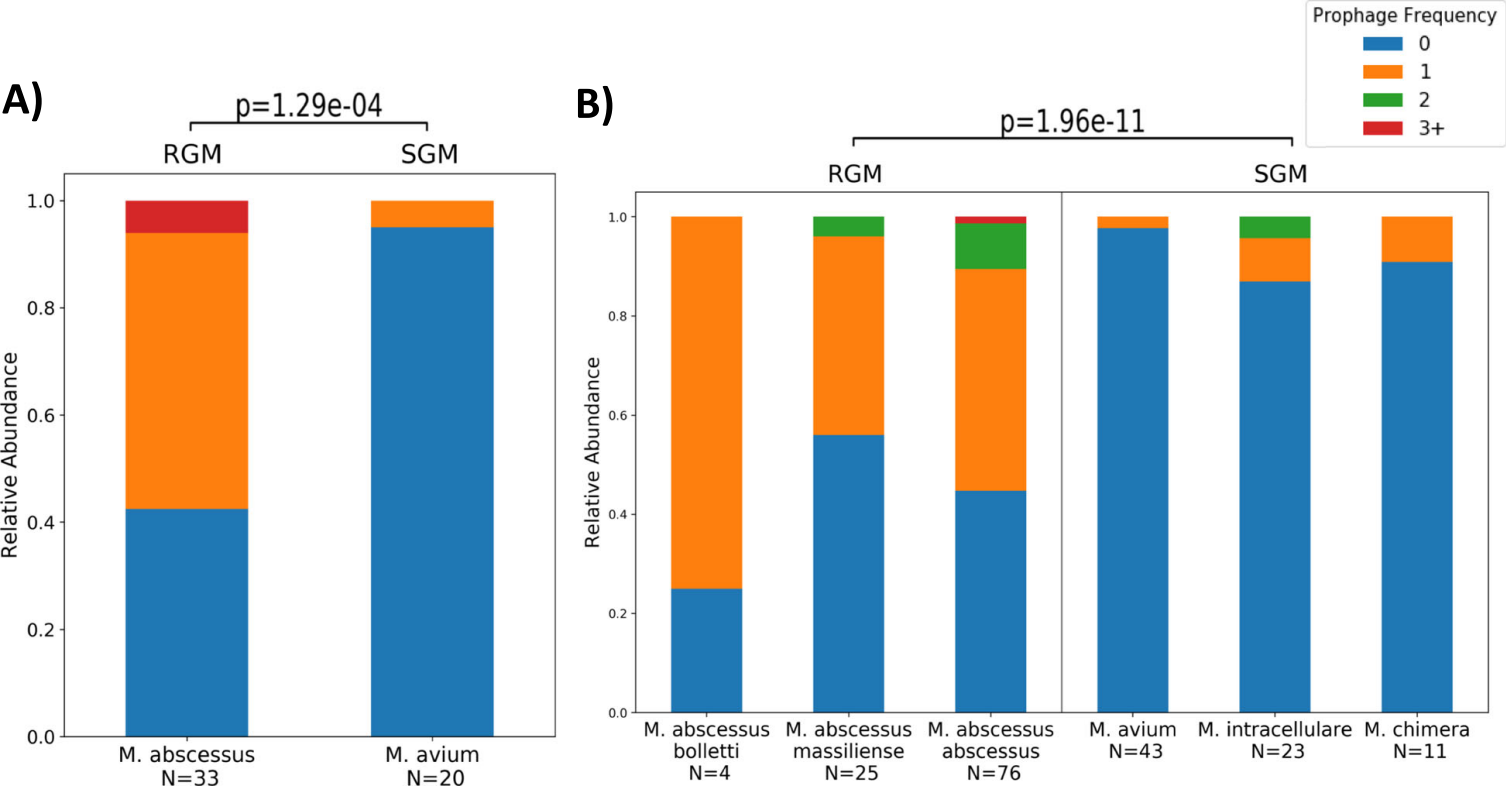
Prophage Frequency by NTM Species: Bar plots show relative abundance of prophage frequency in samples. Rapidly growing mycobacteria species are on the left, and slowly growing mycobacteria species are on the right. **a**) The frequency of prophages by genome in complete NTM genomes. The presence of prophages is statistically significant by growth rate (*p*  =  1.96e-11). **b**) The frequency of prophages per draft genome from NTM draft genomes. The presence of prophages is statistically significant by growth rate (*p*  =  1.29e-04)

Prophages were predicted in 81 of the 235 clinical NTM genomes (37.7% of complete genomes and 33.5% of draft genomes). Within the 81 genomes with prophages, a total of 96 unique prophages were identified. The interquartile range of prophage lengths across all species was ~ 38 kilobases to ~ 58 kilobases. A Kruskal-Wallis test of prophage lengths by growth rate failed to reject the null hypothesis suggesting no difference in the size of integrated prophages by growth rate (H = 1.93, *p =* 0.165). We predicted integrated prophage elements in both RGM and SGM, however predicted prophages more likely to be found within RGM than in SGM (F  =  6.71, *p*  =  1.96e-11 draft genomes and F  =  3.83, *p*  =  1.29e-04 complete genomes, proportions z-test, Fig. 1). In the RGM *M. abscessus* subsp. *abscessus,* 61 out of 109 (56.0%) clinical NTM have predicted prophage elements, while of the SGM, only 2 out of 63 *M. avium* (3.2%), 3 out of 23 *M. intracellulare* (13%) and 1 out of 11 *M. chimaera* (9.1%) draft genomes have intact prophage elements. The relative genomic locations of the predicted prophages within the complete genome dataset are shown in Supplementary Figure 1.

To test if the prophage discovery tools are biasing prophage predictions in longer contiguous sequences (i.e. complete genomes), we quantified the number of edge cases by species in the 182 draft genomes. The number of edge cases are normalized by genome count per species to generate an average number of edge cases per species (Table 1). Edge cases are only quantified in the draft genome assemblies because the complete genomes are assumed to have only one contiguous sequence (Table 2). Linear mixed models of prophage frequency by number of contigs (Z = -3.27, *p =* 1.1e-03), N50 Length (Z = 1.94, *p =* 0.052), and number of contigs > 1500 base pairs (Z = -4.10, *p =* 4.19e-04) approached or achieved significance. However, additional post-hoc testing of significant linear mixed models revealed no significant correlations suggesting assembly fragmentation likely had little effect on the number of predicted prophages between species.

**Table 2:** Summary statistics of predicted prophages within complete NTM genomes selected from the NCBI assembly database. Complete genomes are not fragmented into contigs, thus N50 statistics, contig count, and edge cases are not applicable. Additional information about these genomes are available in Supplementary File 1

Species	Genomes Count	Genomes with Phage	Predicted Prophages	Median tRNA Count
*M. avium*	20	1	1	59 (54–59)
M. abscessus	33	19	23	49 (46–104)

Thus, in future figures, draft and complete genomes are combined for downstream analysis.

### Predicted prophages annotated using existing mycobacteriophage clusters

**Fig. 2:**
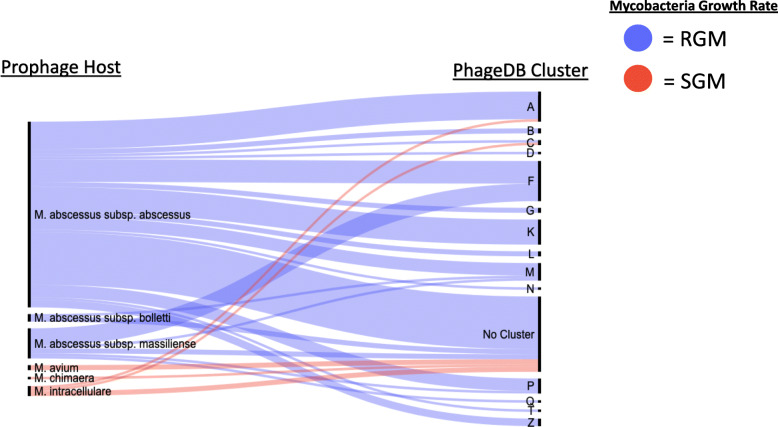
Prophage Assignments to PhagesDB Clusters: Alluvial graph depicting assignment of predicted prophages by NTM species to a PhagesDB lettered cluster (on the right) and NTM species (on the left). Line width corresponds to the number of predicted prophages from a genome that are assigned to a specific PhagesDB cluster

PhagesDB is a database containing mycobacteriophage genomes categorized into clusters and sub clusters based on sequence similarity and shared functional genes. Predicted prophages in our study were annotated across 15 clusters (Fig. 2) using ANI and protein sequence similarity. The RGM had prophages in all 15 lettered groups, and SGM had 3 lettered groups. Most prophages (31.3%) fell into the “no cluster” category, which represents prophages matching other prophages without a defined cluster or not being assigned a cluster using ANI or BLASTp. The number of errors associated with the ANI assignment to lytic clusters is on average nearly double that of prophages assigned to lysogenic clusters (22,171 lytic against 12,385 lysogenic base pair errors, *p* = 0.098, Mann Whitney U).

Across the entire dataset (draft and complete genomes), the 96 identified prophages consisted of 84 genotypically unique sequences. Predicted prophages that clustered with another predicted prophage were from the same species, which could represent a shared evolutionary sequence or a common insertion site.

The pangenome analysis using all predicted prophages resulted in no core genes shared among 96 unique prophages. The largest number of prophages that shared a gene was 25 prophages and the gene function was undefined. Supplementary Table 1 details the pangenome analyses, including shell gene counts between prophages discretized by assigned PhagesDB cluster. No core genes are found in any lettered cluster pangenome analysis. Hypothetical proteins of unknown function are most commonly shared shell genes within the predicted prophages.

### Prophage annotation results and virulence genes by species

There was an average of 68 open reading frames (ORFs) predicted within each of the 96 prophages from the draft and complete genomes. The average peptide length of ORFs was 216 (24–1937) amino acids. The total number of ORFs among all prophages was 6550. Of these predicted ORFs, 65.24% were labeled hypothetical proteins without a known function. Almost half of the ORFs (48.89%) were annotated using sequence similarity to a protein within the PhagesDB mycobacteriophage database. ARAGORN identified 156 tRNAs with tRNA-threonine as the most abundant, comprising 16.0% of the total. The number of ORFs annotated as bacterial genes in the dataset, not including tRNAs, was 245 (3.74%). Of ORFs identified as bacterial genes, 66 ORFs (1.01% of all ORFs) were predicted to derive from bacterial virulence genes.

**Fig. 3:**
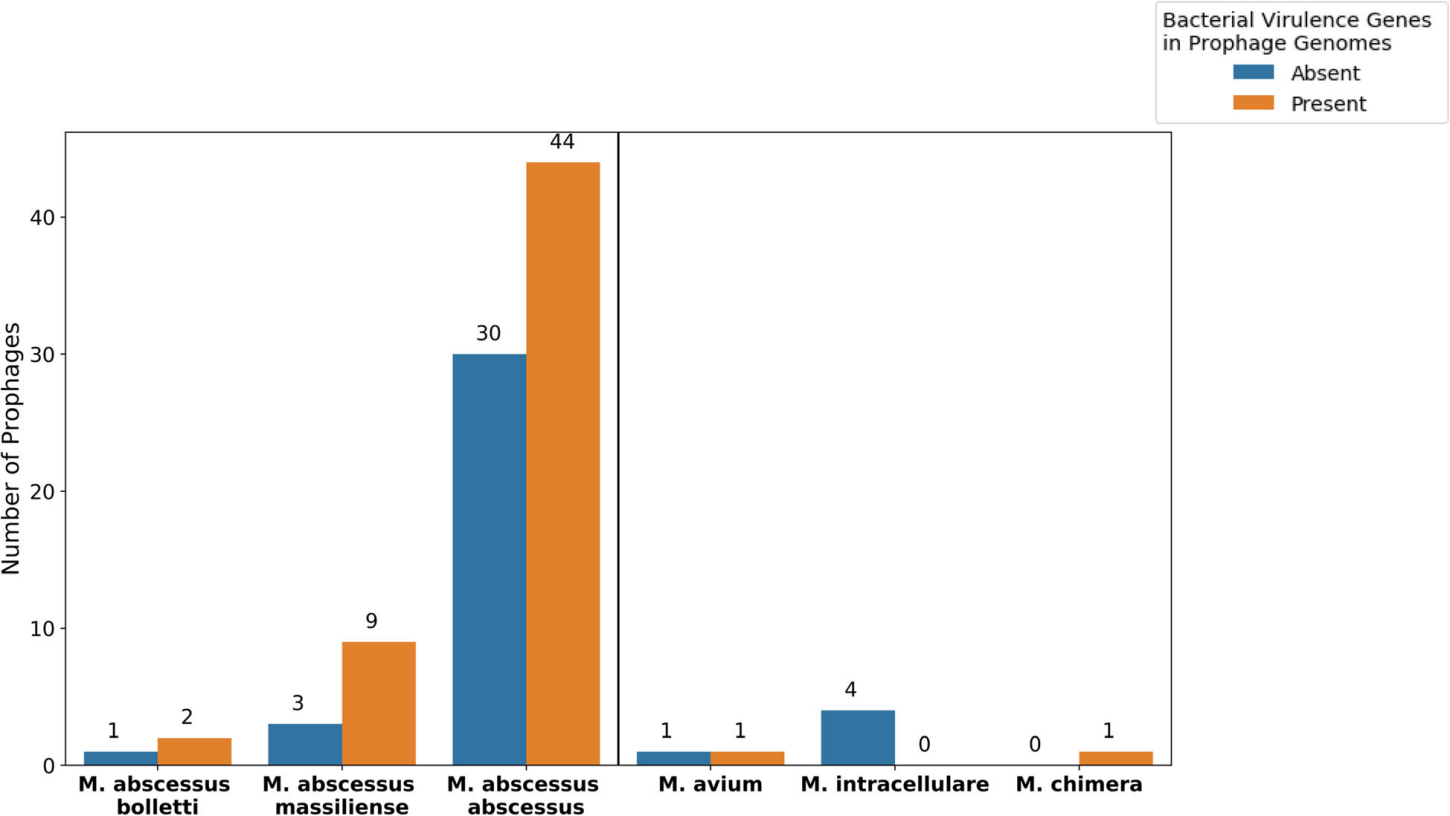
Bacterial Virulence Frequency in Prophages by Species: Bar plots showing the frequency of a bacterial virulence genes within predicted prophages by mycobacterial species. The presence of bacterial virulence genes in prophage genomes is not statistically significant by growth rate (*p =* 0.085)

Virulence gene annotations within the predicted prophages comprised about 1% of the ORFs. The 66 predicted ORFs annotated as bacterial virulence genes were present across 39 prophages from 37 different NTM genomes. Figure 3 highlights the presence/absence of bacterial virulence gene within the predicted prophages across mycobacterial species. Of the genes annotated as virulence genes by VFDB or Patric-VF, 51.5% were originally identified in *Mycobacterium tuberculosis* and 16.7% derive from *Salmonella enterica* (Supplementary File 2)*.*

**Fig. 4:**
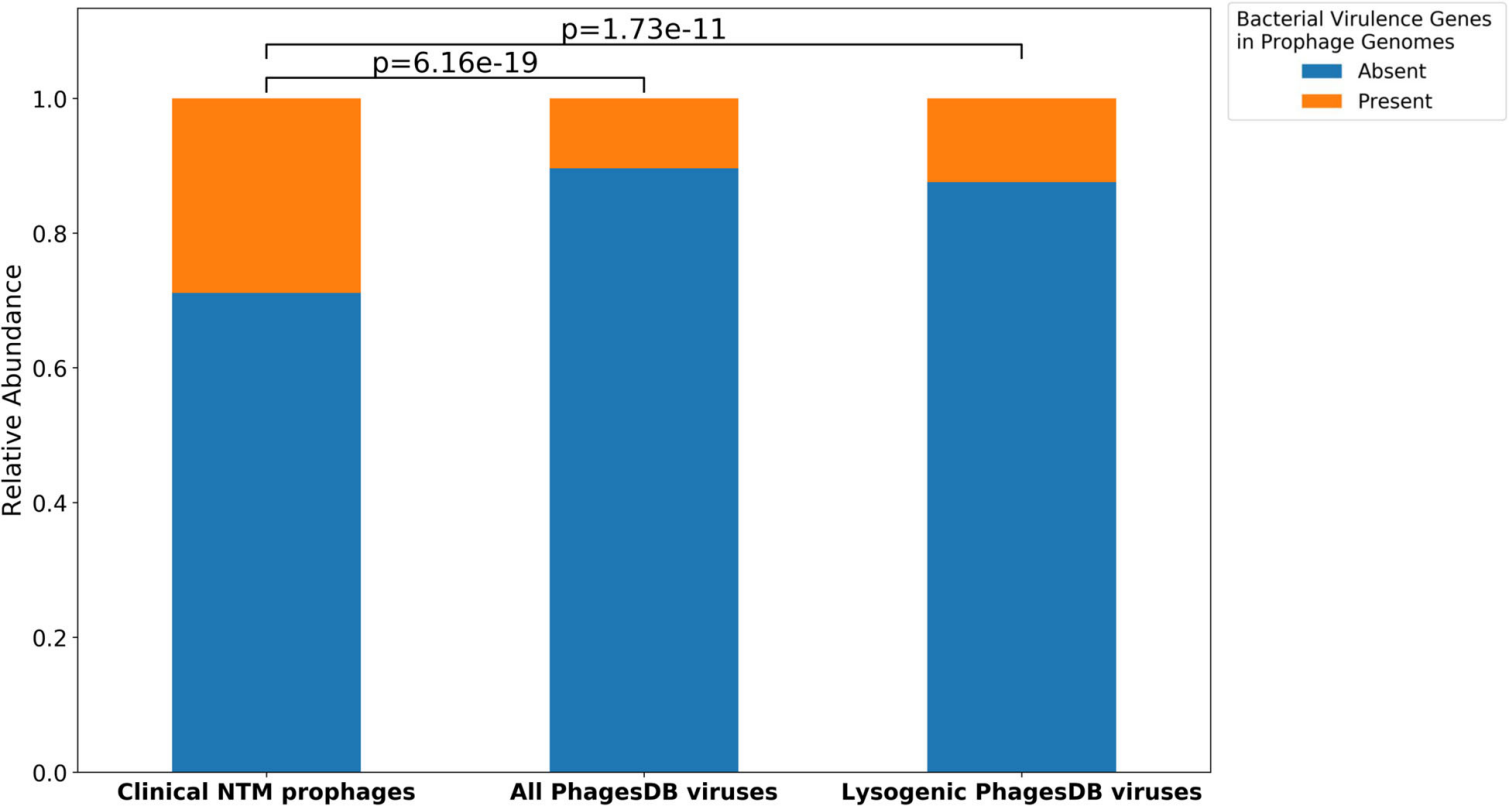
Bacterial Virulence Frequency in Prophages by Data Source: Bar plots showing relative abundance of bacterial virulence genes within viral genomes from our predicted prophages, mycobacteriophages from PhagesDB, and lysogenic mycobacteriophages of PhagesDB. The presence of bacterial virulence genes in the genomes of our predicted prophages is statistically significant against the presence of bacterial virulence genes in both the full PhagesDB mycobacteriophages and the lysogenic PhagesDB mycobacteriophages (F  =  8.89, *p*  =  6.16e-19 and F  =  6.73, *p*  =  1.73e-11, proportions z-test)

**Fig. 5:**
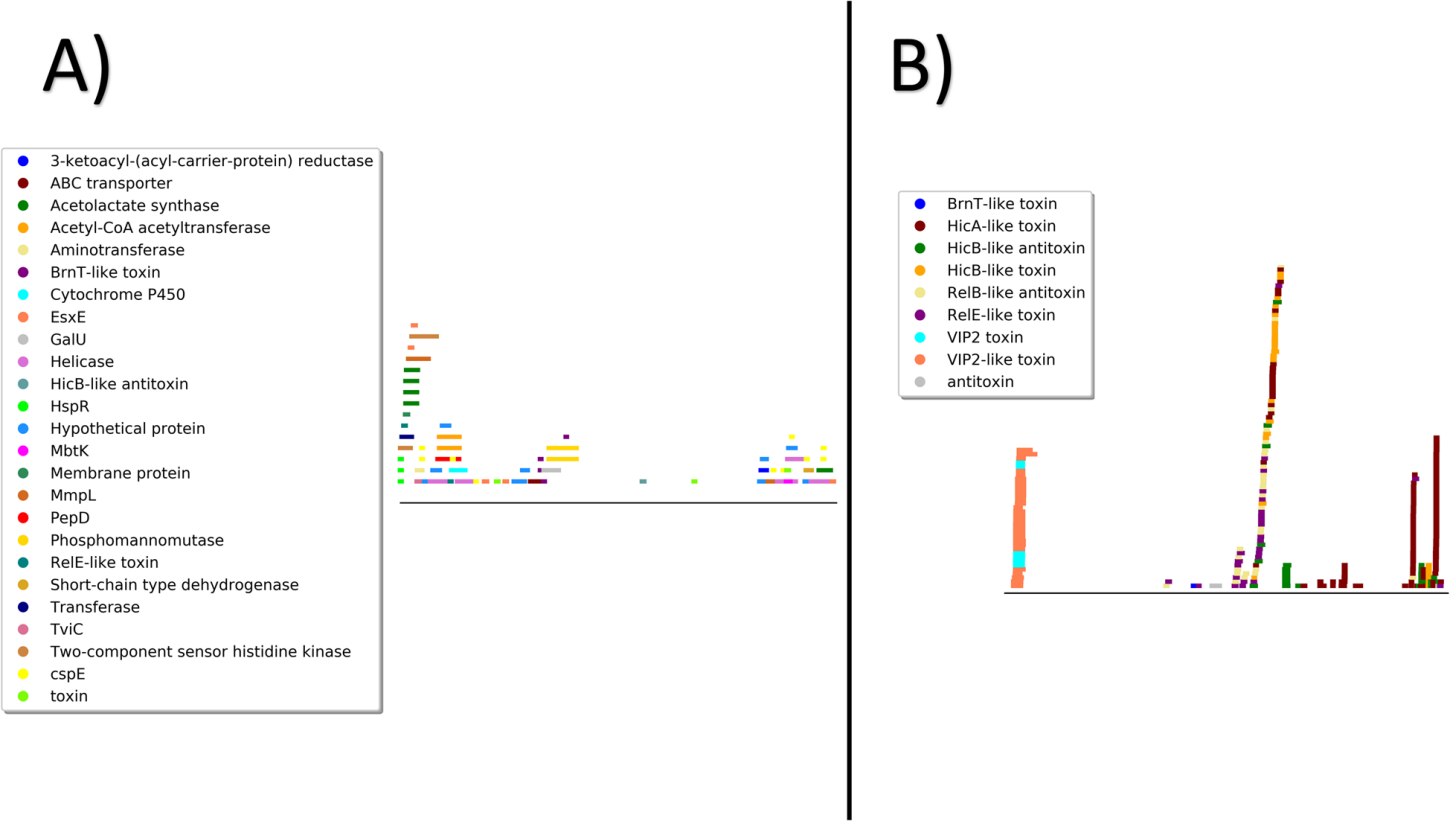
Relative Location of Bacterial Virulence Genes: Line graph showing relative abundance, location, and annotation of bacterial virulence genes within viral genomes from our predicted prophages and mycobacteriophages from PhagesDB. The presence of bacterial virulence genes in the genomes of our predicted prophages is statistically significant against the presence of bacterial virulence genes in both the full PhagesDB mycobacteriophages (F  =  8.89, *p*  =  6.16e-19 proportions z-test). **a**) Bacterial virulence genes in predicted prophages (*n* = 66). **b**) Bacterial virulence genes in mycobacteriophages from PhagesDB (*n* = 249)

PhagesDB contains 1795 mycobacteriophages. Within these mycobacteriophages, 187 mycobacteriophages contained 249 virulence genes or 0.13% of all predicted ORFs. For a fairer comparison of virulence gene abundance between our predicted prophages and PhagesDB, lysogenic mycobacteriophages were subset from PhagesDB leaving 1271 mycobacteriophages and 122,405 ORFs. Within these 1271 mycobacteriophages, 158 mycobacteriophages contain 220 annotated virulence genes (0.18% of all ORFs). We found that bacterial virulence genes are more likely to be present within prophage elements derived from clinical sources than from all mycobacteriophage genomes in PhagesDB isolated from the environmental M. *smegmatis* and the subset selected from lysogenic mycobacteriophages (F  =  8.89, *p*  =  6.16e-19 and F  =  6.73, *p*  =  1.73e-11, proportions z-test, Fig. 4). The relative locations and functional annotations of bacterial virulence genes in the predicted prophages, as well as PhagesDB mycobacteriophages are shown in Fig. 5. The location of the virulence genes in the predicted prophages, with 43.9% within 10% of either end of the predicted prophage, suggests either specialized transduction, where genes flanking the prophage insertion site are transduced or an error in the predicted prophage range (Fig. 5). 125 bacterial virulence genes (50.2%) are within 10% of mycobacteriophage ends in the PhagesDB database (Fig. 5). The presence of virulence genes in clinical isolates is significantly enriched even when these virulence genes near the ends are removed from only the predicted prophages (F  =  5.03, *p*  =  4.85e-7 and F  =  3.37, *p*  =  7.44e-4, proportions z-test).

### Mycobacterium phylogeny influences on prophage frequency

To test if NTM draft genomes with prophages are more evolutionarily similar to each other than to NTM draft genomes without prophage, we performed a PERMANOVA test of phylogenetic distance metrics. The genome wide genetic distances of *M. abscessus* subsp*. abscessus* draft genomes are more similar within the groups: with and without prophages, than between groups (F  =  2.17, *p*  =  0.026, PERMANOVA). Supplementary Figure 2 displays the distribution of prophages in M. *abscessus subsp. abscessus* and M. *abscessus subsp. bolletti* in the context of the bacterial phylogeny. The distributions of predicted prophages in other species are shown in Supplementary Figures 3 and 4.

CRISPR elements have the capability to protect a bacterium against prophage integration. CRISPR elements were present in only 4 of the 53 (7.5%) complete genomes and 12 of the 182 (6.6%) draft genomes. Presence of CRISPR elements was not associated with the number of prophages (H = 0.0092, *p =* 0.92, Kruskal-Wallis). The abundance of tRNAs in the mycobacterial genomes was significantly different by growth rate with slowly growing mycobacterial species having a greater number of tRNAs (H = 89.43, *p =* 3.18e-21, Kruskal-Wallis). In addition, the relationship of tRNAs and prophage frequency corrected by species reveals a positive linear correlation only in *M. abscessus* (R^2^ = 0.34, *p =* 3.21e-4).

## Discussion

In this study, we detail the frequency of prophages within six different species of NTM from 182 draft genomes and 53 complete genomes. Prophages in this study are predicted using an ensemble approach combining two tools that identify prophages in different ways [60]. Prophage prediction in this study does not guarantee viruses capable of excising, but is indicative of integrated elements that contribute to the evolution of the host.

The number of prophages found in this study within mycobacteria with a rapid growth rate is greater relative to the number found in slowly growing mycobacteria (Fig. 1). Assemblies of SGM are more GC rich than RGM, which is correlated with higher contig numbers (Table 1). Prophage identification does not appear to be driven by assembly fragmentation as evident by the edge case ratio. A higher edge case ratio in RGM compared to SGM means that if prophages are missing from the assembly they are more likely to be missing in RGM. In addition, the correlation between the number of contigs, median contig length, number of sequences > 1500 base pairs, and the number of predicted prophages did not hold during post-hoc testing. This further supports the notion that assembly fragmentation is not affecting the identification of prophages.

Our analyses revealed that the presence of CRISPR elements are rare in NTM, as only 16 of 235 samples (6.8%) contained CRISPR elements. Prior studies of CRISPR elements in mycobacteria found loci with greater than 5 repeats only in *M. tuberculosis*, *M.bovis,* and *M. avium* species [61]. Prophage frequency did not correlate with the presence of CRISPR elements, although the sample size of mycobacteria with CRISPR elements could be a limiting factor within this study.

tRNAs can act as an insertion site target for prophages using a tyrosine-integrase [21]. Our hypothesis was that an increased abundance of tRNA would result in more prophages due to the increase in potential target integration sites for tyrosine-integrases in mycobacteriophages. The abundance of tRNAs in SGM is enriched relative to RGM. This is counterintuitive to our hypothesis considering the frequency of prophages by growth rate. We did observe a positive correlation of tRNA counts and prophages within *M. abscessus* subsp. *abscessus* genomes though our sample size is limited for draft genomes with higher numbers of prophages. Interestingly, the increased abundance of tRNAs in SGM did not translate to increased frequency of prophages compared to RGM, which may be a result of prophages using a different integrase or SGM inhibiting the integration of prophages using another mechanism [22].

The evolutionary advantage for RGM to have more prophages is unclear in the context of this study. Prior studies in *E. coli* have shown an increased growth rate linearly corresponds to an increase in burst size of phages from the host [62]. Additionally, the increased abundance of host bacteria may achieve a threshold necessary for bacteriophage replication thus allowing the continuation of integrated prophages in subsequent colonies of RGM [63]. *M. smegmatis*, the nonpathogenic model organism that is commonly used to isolate mycobacteriophages is a RGM [27].

Many predicted prophages did not share significant similarity to other known mycobacteriophages. The low similarity between the predicted prophages and the known mycobacteriophages could be a result of genetic mosaicism or genetic degradation of the integrated element. Of those that were assigned to a cluster, 12 prophages were assigned to clusters by ANI that typically exhibit a lytic life cycle. Changes to the threshold of amount errors allowed in cluster assignment may affect the clustering of predicted prophages. The prophages predicted in this study do not share a core genome reflecting the wide variability of viruses. The gene shared amongst the most prophages was present in only 25 prophages. This gene and many others that are highly shared have no defined function and are labeled hypothetical proteins. Of note, the high mosaicism found in this study supports prior explorations of mycobacteriophage genomes. Pedulla et al. annotated 10 novel mycobacteriophages and noted the abundance of previously defined bacterial genes within viral genomes (3.1% of ORFs) including those homologs with the potential to elicit an immune response in humans against *M. tuberculosis* and *M. leprae* [64].

Annotations of the predicted prophage elements supports the rarity of transduction events, with 3.74% of ORFs predicted annotated as bacterial genes. Also, virulence genes were more abundant within prophages from clinical NTM genomes than environmental mycobacteriophages cataloged in PhagesDB (1.01% clinical NTM vs 0.13% PhagesDB). Our results support our hypothesis suggesting prophages could act as a reservoir of bacterial genes important for virulence (Fig. 4). The location of the virulence genes near the ends of predicted prophages suggest either specialized transduction, where genes flanking the prophage insertion site are transduced or an error in the predicted prophage range (Fig. 5). The percentage of virulence genes identified near the ends of prophages and mycobacteriophages is similar (43.9% in prophages and 50.2% in mycobacteriophages) and removing the virulence genes on the ends from the predicted prophages maintained the observation that bacterial virulence genes are more likely to be present within prophage elements derived from clinical sources than from mycobacteriophages of PhagesDB.

Though clinical NTM are known to display different levels of virulence, even within a species, it is unclear if virulence genes within prophage elements affect patient outcomes [65]. Presence of virulence genes alone does not mean these genes are actively expressed, and the presence of a prophage in a genome does not guarantee a functional or excisable virus. Further studies of RNA transcription of mycobacteria with prophages would be helpful in characterizing the expression of phage genes. In addition, our study relied on PhagesDB as an environmental proxy of mycobacteriophage. Future studies exploring prophage frequency within environmental isolates of mycobacteria are needed to directly compare prophage susceptibility of clinical and environmental NTM genomes.

This study demonstrates the presence of prophages in clinical species of mycobacteria. Prophages offer a mechanism for the genetic mosaicism of mycobacteria which have been observed to lack a distributed conjugal transfer (DCT) protein [66]. An increase in the genetic fluidity of a bacterial infection by prophage elements can impact patient outcomes as seen in other pathogens [67]. Mycobacteriophages may contribute to the pathogenic potential of environmental mycobacteria by acting as an external genetic reservoir. Additional work is needed to understand the role of mycobacteriophages in shaping the dynamics of mycobacterial infections.

## Conclusions

In summary, our results indicate that prophages are present in the genomes of clinical mycobacteria. Prophages are more likely to be present in mycobacteria with a rapid growth rate compared to slowly growing species. The mechanism and selective advantage of this enrichment by growth rate remains unclear. Prophages within mycobacteria do not share a core genome and are genetically distinct. Comparisons to other mycobacteriophages from PhagesDB revealed some similarities, including shared members of lettered clusters, however the largest group of integrated prophages were not assigned to a previously defined cluster. In addition, bacterial virulence genes were enriched in predicted prophages from clinical genomes relative to environmental mycobacteriophages from PhagesDB. Our comprehensive analysis of prophage frequency and their genetic composition provides insight into the capability of mycobacteriophages to transduce bacterial genes relevant to bacterial virulence, potentially influencing the progression of disease.

## Supplementary information

**Additional file 1: Table S1*.*** Abundance of shell genes in prophages calculated from pangenome analysis using Roary. Shell genes are defined as genes present in 15 to 95% of genomes in a cluster. This analysis of prophages assigned to lettered clusters was only applied to clusters with 5 or more prophages.

**Additional file 2.** This file contains the RefSeq assembly accession, NCBI Assembly ID, Bioproject, species, and source of the complete genomes used in this study.

**Additional file 3.** This file contains 6 data tables used in the study. The Predicted_Prophages tab provides details about the predicted prophages including the genomic coordinates, cluster assignment, and the presence/absence of a bacterial virulence gene. The Predicted_Prophages_Edge_Cases lists the coordinates of prophages near the ends of contigs. The Bacterial_Genes tab represents the annotated output of bacterial genes (excluding bacterial virulence genes). The Virulence_Genes represents the annotated output of bacterial virulence genes. The Cluster_Virulence tab groups the predicted prophages by cluster and virulence status. The Virulence_Gene_Origins tab represents a count of the species origin of a virulence gene in the predicted prophages.

**Additional file 4: Figure S1.** Relative locations of predicted prophages in *M. absessus subsp. absessus* and *M. avium*. Prophages are colored by the host origin.

**Additional file 5: Figure S2.** Genome wide phylogeny of 80 *M. abscessus* subsp*. abscessus* and *M. abscessus* subsp. *boletti* genomes and 5 control genomes. The heatmap on the right shows the presence (black) or absence (white) of the lettered PhagesDB clusters (x-axis). The presence of prophage in a sample is noted by a shaded box in any column except NA/Control. NA/Control shading signifies genomes without a prophage and added controls not analyzed for prophages in this study. No shading means the sample does not have a prophage in that lettered cluster.

**Additional file 6: Figure S3.** Phylogeny of host *M. absessus subsp. massiliense* genomes. The shaded boxes are located along an x axis, which lists the lettered PhagesDB clusters. The presence of prophage in a sample is noted by a shaded box in any column except NA/Control. No shading means the sample does not have a prophage in that lettered cluster. Statistical significance was not achieved using PERMANOVA on this tree (F = 1.398, *p =* 0.225).

**Additional file 7: Figure S4.** Phylogeny of host M*. chimaera* and M*. intracellulare* genomes. The shaded boxes are located along an x axis, which lists the lettered PhagesDB clusters. The presence of prophage in a sample is noted by a shaded box in any column except NA/Control. No shading means the sample does not have a prophage in that lettered cluster. Statistical significance was not achieved using PERMANOVA on this tree (F = 1.12, *p =* 0.321).

## Data Availability

All scripts used to derive the figures and additional preprocessing including tool overlap identification are available on the Strong Lab GitHub at https://github.com/Strong-Lab/Prophage_In_NTM. The fasta sequences of predicted prophages are available on the Github at https://github.com/Strong-Lab/Prophage_in_NTM/tree/master/data/fasta. The raw data of the clinical NTM draft genomes are available at BioProject 319839 and the sources of the complete genomes are listed in Supplemental File 1. Additional data tables are included in Supplementary File 2.

## Abbreviations

NTM: Nontuberculous mycobacteria
RGM: Rapidly growing mycobacteria
SGM: Slowly growing mycobacteria
MAC: *M. avium* complex
PATRIC-VF: Pathosystems Resource Integration Center virulence factor database
VFDB: Virulence Factor Database
CRISPR: Clustered regularly interspaced short palindromic repeats
ANI: Average nucleotide identity
RAxML: Randomized Axelerated Maximum Likelihood-Next Generation
DCT: Distributed conjugal transfer
