# Supplementary material for: Characterization of integrated prophages within diverse species of clinical nontuberculous mycobacteria
Source: Virol J. 2020 Aug 17;17:124. doi: 10.1186/s12985-020-01394-y (PMC7433156; doi:10.1186/s12985-020-01394-y)
Supplement: Supplementary file 1 — Additional file 1: Table S1. Abundance of shell genes in prophages calculated from pangenome analysis using Roary. Shell genes are defined as genes present in 15 to 95% of genomes in a cluster. This analysis of prophages assigned to lettered clusters was only applied to clusters with 5 or more prophages. [file 12985_2020_1394_MOESM1_ESM.docx]

| **Lettered PhagesDB Cluster** | **Number of Prophages** | **Shell Gene Count** |
| --- | --- | --- |
| A | 12 | 57 |
| F | 16 | 96 |
| K | 10 | 137 |
| M | 7 | 66 |
| P | 6 | 217 |
| No Cluster | 30 | 46 |

Supplementary Table 1: Abundance of shell genes in prophages calculated from pangenome analysis using Roary. Shell genes are defined as genes present in 15% to 95% of genomes in a cluster. This analysis of prophages assigned to lettered clusters was only applied to clusters with 5 or more prophages.
