# Supplementary figures and images for: Characterization of integrated prophages within diverse species of clinical nontuberculous mycobacteria
Source: Virol J. 2020 Aug 17;17:124. doi: 10.1186/s12985-020-01394-y (PMC7433156; doi:10.1186/s12985-020-01394-y)

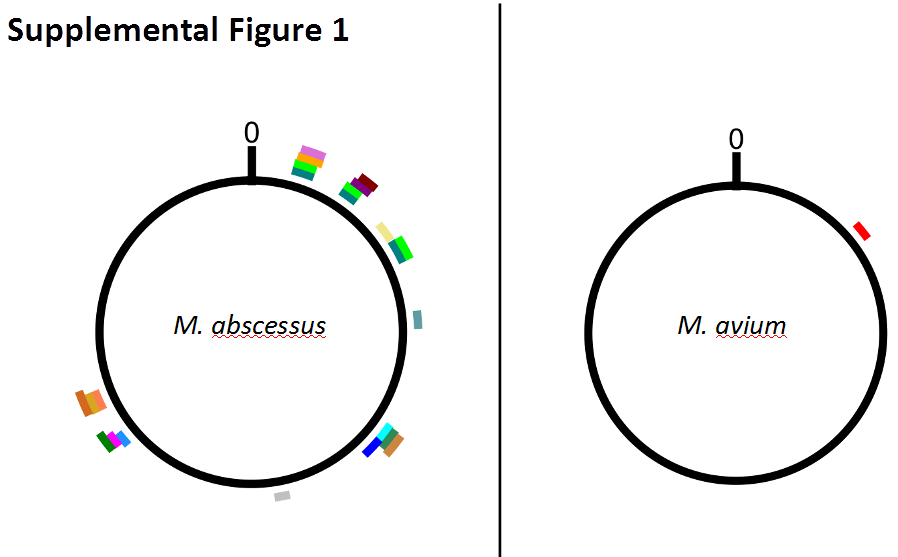

Supplement: Supplementary file 4 — Additional file 4: Figure S1. Relative locations of predicted prophages in M. absessus subsp. absessus and M. avium. Prophages are colored by the host origin. [file 12985_2020_1394_MOESM4_ESM.docx]
