# Supplementary material for: Characterization of integrated prophages within diverse species of clinical nontuberculous mycobacteria
Source: Virol J. 2020 Aug 17;17:124. doi: 10.1186/s12985-020-01394-y (PMC7433156; doi:10.1186/s12985-020-01394-y)

Supplementary Figure 4

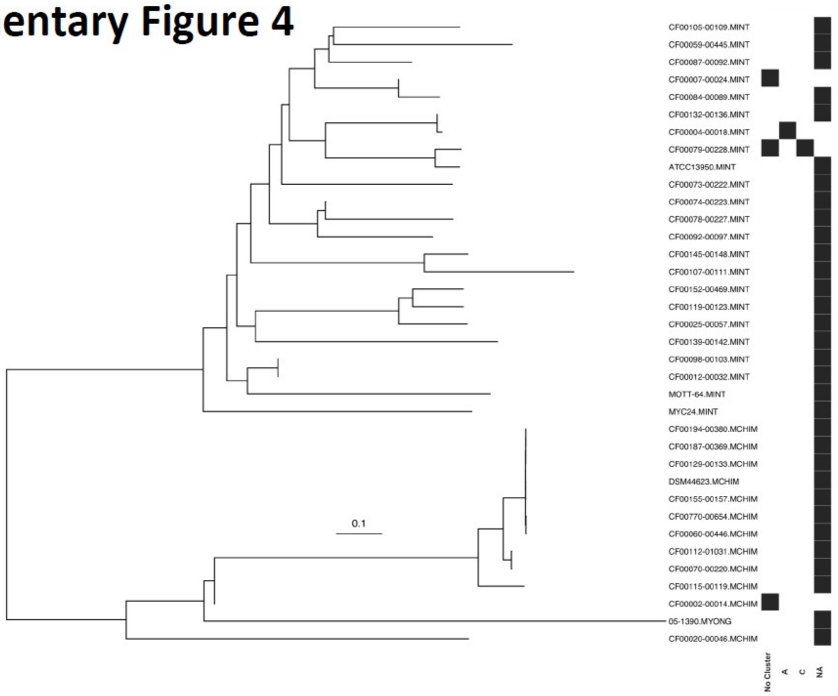

Supplement: Supplementary file 6 [file 12985_2020_1394_MOESM7_ESM.docx]
